# Gastro-oesophageal reflux disease increases the risk of intensive care unit admittance and mechanical ventilation use among patients with chronic obstructive pulmonary disease: a nationwide population-based cohort study

**DOI:** 10.1186/s13054-015-0849-1

**Published:** 2015-03-24

**Authors:** Chen-Liang Tsai, Yu-Huei Lin, Meng-Ting Wang, Li-Nien Chien, Chii Jeng, Chih-Feng Chian, Wann-Cherng Perng, Chi-Huei Chiang, Hung-Yi Chiou

**Affiliations:** Division of Pulmonary and Critical Care, Tri-Service General Hospital, National Defense Medical Center, No. 325 sec. 2 Chenggong Road, Taipei, 114 Taiwan; Graduate Institute of Nursing, College of Nursing, Taipei Medical University, No. 250 Wu-Hsing Street, Taipei, 110 Taiwan; School of Pharmacy, National Defense Medical Center, No. 161, Minguan E. Road, Taipei, 114 Taiwan; School of Health Care and Administration, College of Public Health and Nutrition, Taipei Medical University, No. 250 Wu-Hsing Street, Taipei, 110 Taiwan; Division of Respiratory Therapy, Chest Department, Taipei Veterans General Hospital, No. 201, Sec. 2, Shipai Road, Taipei, 112 Taiwan; School of Public Health, College of Public Health and Nutrition, Taipei Medical University, No. 250 Wu-Hsing Street, Taipei, 110 Taiwan

## Abstract

**Introduction:**

Gastro-oesophageal reflux disease (GORD) is common among chronic obstructive pulmonary disease (COPD) patients and may have a deleterious effect on COPD prognosis. However, few studies have investigated whether GORD increases the risk of severe outcomes such as intensive care unit (ICU) admittance or mechanical ventilator use among COPD patients.

**Methods:**

Propensity score matching by age, sex, comorbidities and COPD severity was used to match the 1,210 COPD patients with GORD sourced in this study to 2,420 COPD patients without GORD. The Kaplan-Meier method was used to explore the incidence of ICU admittance and machine ventilation with the log rank test being used to test for differences. Cox regression analysis was used to explore the risk of ICU admittance and mechanical ventilation use for patients with and without GORD.

**Results:**

During the 12-month follow-up, GORD patients and non-GORD patients had 5.22 and 3.01 ICU admittances per 1000 person-months, and 4.34 and 2.41 mechanical ventilation uses per 1000 person-month, respectively. The log rank test revealed a difference in the incidence of ICU admittance and machine ventilation between the two cohorts. GORD was found to be an independent predicator of ICU admittance (adjusted hazard ratio (HR_adj_) 1.75, 95% confidence interval (CI) 1.28-2.38) and mechanical ventilation (HR_adj_ 1.92, 95% CI 1.35-2.72).

**Conclusion:**

This is the first investigation to detect a significantly higher incidence rate and independently increased risk of admission to an ICU and mechanical ventilation use among COPD patients who subsequently developed GORD during the first year following their GORD diagnosis than COPD patients who did not develop GORD.

**Electronic supplementary material:**

The online version of this article (doi:10.1186/s13054-015-0849-1) contains supplementary material, which is available to authorized users.

## Introduction

Chronic obstructive pulmonary disease (COPD) is a major problem worldwide and a risk factor for increased mortality and morbidity [1-3]. The vast majority of unscheduled visits and hospitalizations for COPD patients can be attributed to acute exacerbations, which are episodes of increased respiratory compromise that contribute to accelerated lung function decline, impaired quality of life, related morbidity and mortality, and increased economic costs [4,5].

To date the etiology of one-third of all acute exacerbations remains unclear [2,5,6]. One putative risk factor that has gained attention over the past decade for these idiopathic exacerbations is gastro-oesophageal reflux disease (GORD). Earlier studies have reported a high prevalence of GORD among COPD patients [7-11], with preliminary data suggesting that gastro-oesophageal reflux may heighten bronchial reactivity in GORD patients through esophago-bronchial reflux and microaspiration [12,13], which in turn may precipitate a greater frequency of COPD exacerbations [11,14-17]. Because many COPD patients are simultaneously afflicted with GORD, and because GORD has been suggested to exacerbate COPD symptoms, GORD may thus represent a novel risk factor for exacerbations that is highly prevalent in the COPD patient population.

Currently, the studies in the literature exploring the association between GORD and COPD exacerbations have all utilized self-reported GORD symptoms and prevalent cases in their analyses [12-14,18]. While these studies did observe an increase in the frequency of acute exacerbations of chronic obstructive pulmonary disease (AECOPD), they did not comment on the extent of the AECOPD event severity. The data available in the literature are therefore insufficient to establish causality, to establish temporality, or to link the effect of GORD to any specific outcome severity among COPD patients.

There is evidence indicating that patients who require mechanical ventilation or admission to an ICU generally have poor prognoses and consume large amounts of healthcare resources, with respiratory failure secondary to COPD being their most common cause of death (56.8%) [19]. Moreover, while the in-hospital mortality of patients suffering from an exacerbation has been reported to range between 10 and 20% [1,20], the mortality rate of patients requiring mechanical ventilation use has been shown to reach 40% during the first year following their discharge [4,20-22]. As mechanical ventilation use and ICU admission are reliable markers of poor COPD prognosis, this study aimed to investigate whether GORD is associated with an independently increased risk of ICU admittance and mechanical ventilation use to better understand the effect of GORD on COPD severity. This investigation was achieved by leveraging the statistical power of a nationwide healthcare database to conduct a cohort study analysis adjusting for a panel of comorbidities predictive of COPD prognosis as well as COPD severity in accordance with Global Initiative for Chronic Obstructive Lung Disease (GOLD) guidelines [23].

## Materials and methods

### Data sources

The information analyzed in this study was sourced from the National Health Insurance Research Database (NHIRD). This database comprises the administrative records of the Taiwan Insurance Program, a compulsory social insurance program that covers over 99% of the 23 million citizens of Taiwan. The Taiwan National Health Research Institute, which manages this dataset, used it to create the Longitudinal Health Insurance Database. The Longitudinal Health Insurance Database is a more manageable dataset consisting of all the original claims data spanning the timeframe between the year 2000 and 2010 for 1 million beneficiaries randomly sampled in 2005. There were no statistically significant differences in age distribution, sex distribution, or healthcare costs between the subjects in the Longitudinal Health Insurance Database and all enrollees of the NHIRD [24].

In this study, we used the inpatient and outpatient databases, the catastrophic illness database, the pharmaceutical prescription database, and the registry for beneficiaries. The registry for beneficiaries contained information regarding subject gender, date of birth, date of outpatient visits (including emergency department visits), date of admission, prescriptions, as well as socioeconomic and neighborhood characteristics. The NHIRD protects the privacy and confidentiality of all beneficiaries and provides health insurance data for research only. This study has been approved by the Institutional Review Board of Taipei Medical University (No. 201407047). Furthermore, individual consent to participate was not required or obtained because the data in the NHIRD that could be used to identify patients or care providers are anonymized by scrambling the data cryptographically before being passed along to the National Health Research Institute for database construction, where the data are scrambled again before being released for research purposes.

### Study design

A retrospective population-based cohort study was conducted on COPD patients with and without a diagnosis of GORD sourced between 1 January 2000 and 31 December 2009. The primary outcome measures were ICU admission and mechanical ventilation use. We hypothesized that GORD would precipitate an increased risk of ICU admittance and mechanical ventilation use during the 1-year follow-up.

### Study patient identification

The pool of subjects eligible for study entry consisted of patients aged ≥40 years who had received two or more COPD diagnoses within 1 year of each other at outpatient visits during which they were also prescribed COPD-related medications between 1 January 2001 and 31 December 2009. The diagnosis of COPD was identified based on International Classification of Diseases, 9th Revision, Clinical Modification (ICD-9-CM) codes 491, 492, and 496, and the COPD-related medications utilized in the above-mentioned inclusion criterion comprised short-acting β_2_-agonists, inhaled long-acting β_2_-agonists, inhaled corticosteroids, inhaled anticholinergics, and theophyllines.

Patients were excluded if they had ever been diagnosed with asthma, lung cancer, ventilator dependence, diseases of the esophagus, dyskinesia of the esophagus, malignant neoplasms of the esophagus, Zollinger–Ellison syndrome, alcohol dependence syndrome, or obesity (for ICD-9-CM code definitions, see Additional file 1).

After the COPD patients conforming to the above criteria were enrolled, they were allocated to either the study cohort or the comparison cohort based on their exposure status. Exposure was defined as having received a GORD diagnosis (ICD-9-CM codes 530.11, 530.81, and 530.10) and having taken GORD-related medication (proton pump inhibitors or H2 antagonists). The index date for subjects in the study cohort was defined as the first date on which the subject received a GORD diagnosis.

However, as the comparison cohort was not diagnosed with GORD, pseudo-diagnostic dates that corresponded to the index date of one of the pools of patients with GORD were randomly assigned according to a prior study [25].

This investigation builds on our prior work demonstrating that newly diagnosed GORD increases the risk of exacerbation [25]. In the present study, we sought to characterize the severity of the downstream COPD exacerbations detected in the prior study. As we originally sought to establish temporality between GORD acquired among COPD patients and an increased risk of exacerbation, we excluded patients who had been diagnosed with GORD prior to having being diagnosed with COPD. In order to investigate the temporality of GORD acquired during COPD and the severity of downstream COPD exacerbations, as well as to maintain comparability between the results of these two studies, we also excluded patients with GORD prior to COPD in the present work.

Moreover, AECOPD events are strong predictors for susceptibility to exacerbation in the following year [14]. However, as this study sought to ascertain the independently increased risk of ICU admission and mechanical ventilation associated with GORD among COPD patients, the inclusion of any subjects who recently suffered from an AECOPD, which may or may not have been more likely to occur on account of GORD, may have introduced an unwanted foreign risk into our estimate. Therefore, while probably resulting in a more conservative estimate, we required all of the included subjects to be in a stable status of the disease, and excluded all subjects who had visited the emergency department or were hospitalized within 1 year due to suffering an AECOPD event prior to the index date.

### Potential risk factors

The comorbidity burden of subjects with stable COPD is an established predictor of mortality [26,27]. Because mortality is often associated with increased admission to an ICU and mechanical ventilation, the comorbidity burden may act as a strong confounder in this study. The Charlson Comorbidity Index (CCI) score was developed to predict mortality among patients with chronic diseases and is currently the most widely used tool to adjust for confounding due to comorbidities in epidemiological studies [28].

The CCI was thus used in this study to adjust for comorbidities, with diseases defined by ICD-9-CM codes. To increase the diagnostic validity of the comorbidities included in this study, comorbidities were only included if they were diagnosed at least twice at either clinic or hospital visits more than 30 days apart. In this study, we adjusted for the comorbidity burden by classifying patients into one of four categories based on the CCI (detailed description shown in Table 1).

**Table 1:** **Demographic characteristic for COPD patients with and without GORD**

**Characteristic**	**With GORD (** ***n*** **= 1,210)**	**Without GORD (** ***n*** **= 2,420)**	***P*** **value**
Male	807 (66.69)	1,621 (66.98)	0.861^a^
Age	63.4 (±12.16)	63 (±12.38)	0.414^a^
Age category			
40 to 50	214 (17.69)	461 (19.05)	0.795^a^
51 to 60	267 (22.07)	516 (21.32)	
61 to 70	309 (25.54)	632 (26.12)	
71 to 80	324 (26.78)	616 (25.45)	
≥ 80	96 (7.93)	195 (8.06)	
CCI category			
0	22 (1.82)	55 (2.27)	0.757^a^
1 to 3	563 (46.53)	1,142 (47.19)	
4 to 6	415 (34.3)	802 (33.14)	
≥ 7	210 (34.3)	421 (17.4)	
Proxy COPD severity	40 (3.31)	79 (3.26)	0.947^a^
COPD medication			
SABA	171 (14.13)	396 (16.36)	0.081
LABA	12 (0.99)	22 (0.91)	0.808
SAMA	157 (12.98)	344 (14.21)	0.307
Theophylline	1,071 (88.51)	2,066 (85.37)	0.009
ICS	42 (3.43)	65 (2.67)	0.187
LAMA	15 (1.24)	26 (1.07)	0.657
Vaccines			
Influenza and pneumococcal	454 (37.52)	890 (36.78)	0.662
Residential area			
Urban	675 (55.79)	1,363 (56.32)	0.888^a^
Suburban	418 (34.55)	817 (33.76)	
Rural	117 (9.67)	240 (9.92)	
Occupation category			
1	325 (26.86)	714 (29.5)	0.098^a^
2	535 (44.21)	1,078 (44.55)	
3	350 (28.93)	628 (25.95)	
Monthly insurance premium ($NT)^b^			
No fee	274 (22.64)	628 (25.95)	0.079^a^
1 to 19,199	332 (27.44)	602 (24.88)	
19,200 to 23,999	400 (33.06)	817 (33.76)	
≥ 24,000	204 (16.86)	373 (15.41)	

Data presented as *n* (%) or mean (± standard deviation). CCI, Charlson Comorbidity Index; COPD, chronic obstructive pulmonary disease; GORD, gastro-oesophageal reflux disease; ICS, inhaled corticosteroids; LABA, long-acting β_2_-agonists; LAMA, long-acting muscarinic antagonists; NT, new Taiwan dollar; SABA, short-acting β_2_-agonists; SAMA, short-acting muscarinic antagonists. ^a^Standard difference. ^b^US$1 ≒ $NT30.

Furthermore, items such as baseline respiratory symptoms, pulmonary function, and supplemental oxygen use were not available in the database. COPD severity was therefore assessed using the GOLD guideline recommendation that long-acting muscarinic antagonists or inhaled corticosteroids combined with long-acting β_2_-agonists should be used in patients with severe and very severe airflow limitations (GOLD severity of airflow obstruction grades 3 and 4) and/or frequent exacerbations (groups C and D in the 2011 update) [23]. In this study, we used these treatment regimens as a proxy indicator of COPD severity.

Residential area, occupational category, and monthly insurance premium were also included in our analysis to better adjust for socioeconomic and neighborhood characteristics. The NHIRD divides residential area into three categories: urban, suburban, and rural. The occupational category is also divided into three categories (detailed description shown in Additional file 2). Monthly insurance premiums for the NHIRD are calculated according to monthly income and are categorized into four levels [29].

### Propensity score model

Matching is often used to reduce selection bias in observational studies [30]. Propensity score methods are increasingly used to reduce or minimize the confounding that frequently occurs in observational studies investigating the effect of a treatment or exposure on an outcome [31]. In propensity score matching, matched sets of exposed and nonexposed subjects are formed by virtue of sharing similar propensity score values. These weighted values essentially describe the risk of the subject for the outcome of interest based on how their baseline characteristics predispose them for that outcome irrespective of the exposure of interest [30].

In the current study, pairs were matched in 1:2 ratio based on their calculated propensity scores. The propensity score was calculated based on the results of a multivariate logistic regression model including a panel of covariates consisting of age, sex, index year of GORD diagnosis, CCI score category, and proxy COPD severity. We used calipers of width equal to 0.2 of the standard deviation of the logit of the propensity score, because this caliper width has been found to be optimal under a variety of scenarios [32].

### Outcome measurement

The outcomes of interest in this study were ICU admission and mechanical ventilation. We used inpatient expenditures and the details of inpatient orders from NHIRD by details of inpatient orders numbers to define ICU admission (03010E, 03011 F, and 03012G) and mechanical ventilation (57001B, 57002B, and 57023B). Follow-up began with the index date and ended on the date of the first instance of the following: ICU admission, mechanical ventilation use, administrative censoring following 12 months of observation, or discontinuation of enrollment from the National Health Insurance program.

### Statistical analysis

Subject characteristics were compared using a chi-squared test for categorical variables and Student’s *t* test for continuous variables as the results of the Kolmogorov–Smirnov test and Levene’s test indicated the data’s normality and homogeneity of variance, respectively. Logistic regression was used to calculate the propensity scores. Standardized differences were computed for each matched variable to examine differences between the exposure and comparison subjects with regard to the propensity score. The Kaplan–Meier method was used to plot the cumulative incidence curves for the two groups, which were compared with the log-rank test. Cox proportional hazards regression models were used to assess the effect of GORD on the risk of ICU admission and mechanical ventilation. Univariate analyses were first conducted for each parameter, with factors significant at the *P* ≤0.05 level then being tested simultaneously in a multivariate analysis with a stepwise regression model. All of the statistical models in this study were tested to ensure that they conformed to the proportional hazards assumption. All analyses were performed using SAS 9.3 software (SAS Institute Inc., Cary, NC, USA) and STATA 12 software (Stata Corp LP, College Station, TX, USA). All statistical tests were two sided, with *P* <0.05 considered to indicate statistical significance.

### Sensitivity analysis

To test the robustness of the main findings, we performed two sensitivity analyses to assess the potential confounding introduced by the exclusion criteria. The first sensitivity analysis comprised the subjects in our original model with the additional inclusion of those subjects that had been diagnosed with GORD prior to COPD. We then treated GORD prior to COPD as a covariate in a survival model and followed-up for ICU admittance and ventilator use. The second sensitivity analysis comprised the subjects in our original model with the additional inclusion of those who had suffered an AECOPD within the 1 year preceding the index date. In this analysis, we treated prior AECOPD as a covariate in the survival model and followed-up for ICU admittance and ventilator events.

## Results

### Baseline characteristics

**Figure 1:**
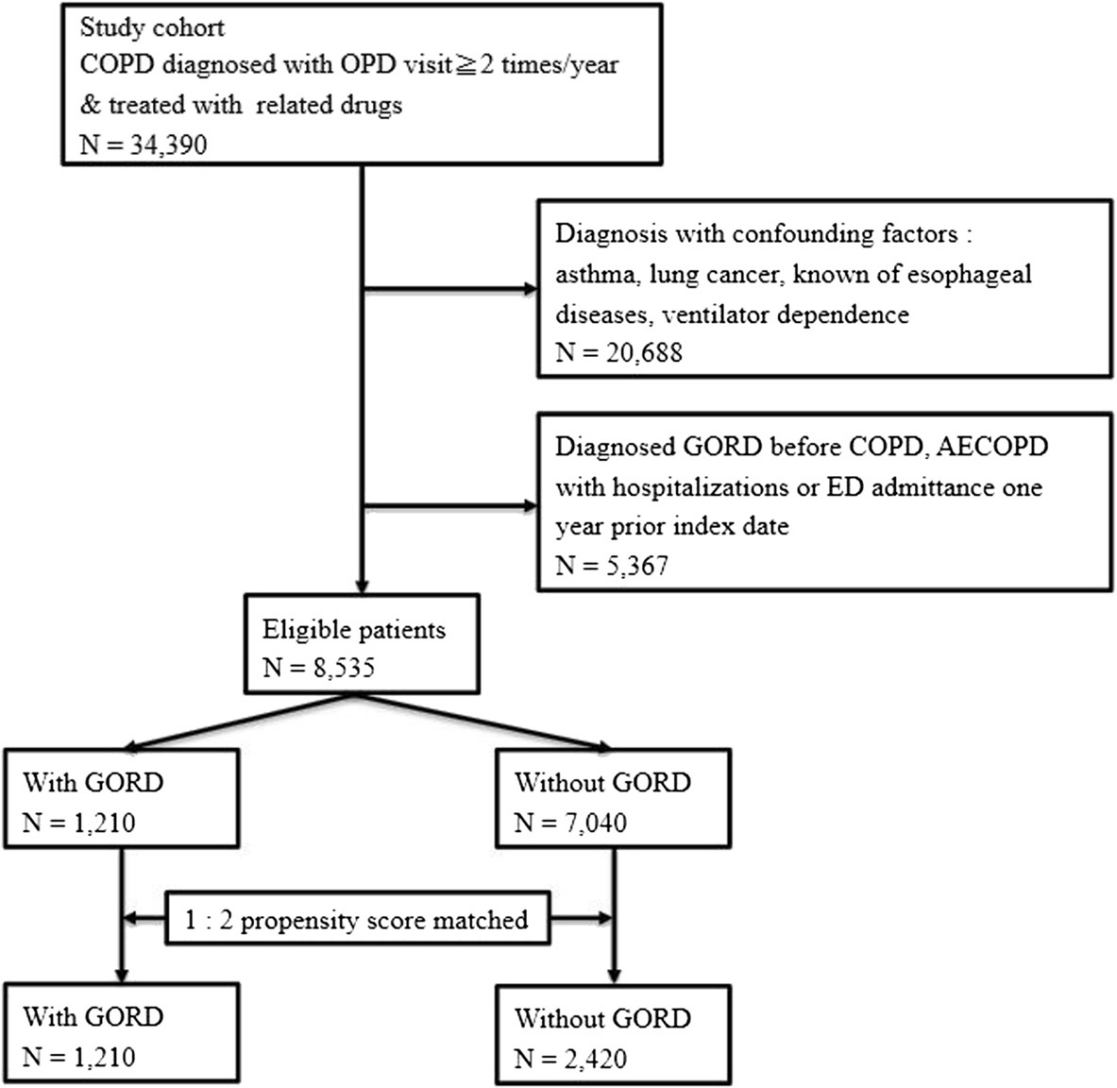
**Flow diagram of the study population.** AECOPD, acute exacerbation of chronic obstructive pulmonary disease; COPD, chronic obstructive pulmonary disease; ED, emergency department; GORD, gastro-oesophageal reflux disease.

After propensity score matching, a total of 1,210 COPD patients with GORD were identified, and 2,420 individuals without GORD were matched (Figure 1). The propensity score provided the discrimination between groups with GORD and without GORD (*C*-statistic, 0.618). Table 1 presents the characteristics of the study patients and the matched comparison subjects. These two groups were quite comparable in terms of sex, age, CCI score category, proxy COPD severity, and socioeconomic status. However, COPD patients with GORD were more likely to have a prescription for theophylline (*P* = 0.009).

### Comparisons of ICU admittance and mechanical ventilator use incidence rates in COPD patients with or without GORD

**Figure 2:**
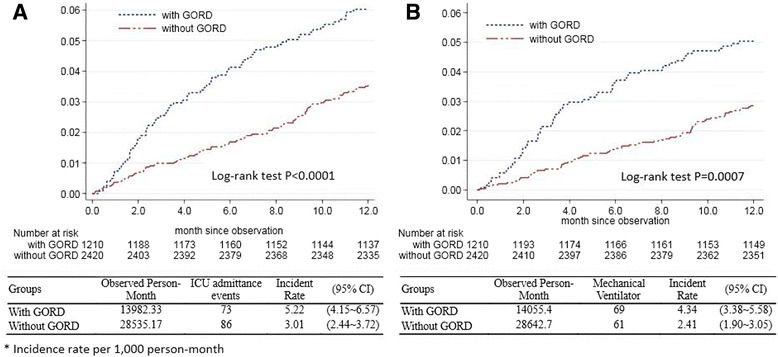
**Cumulative incidence of ICU admittance and ventilator among chronic obstructive pulmonary disease patients with and without gastro-esophageal reflux disease over 12 months. (A)** Incidence rate of ICU admittance. **(B)** Incidence rate of ventilator use. CI, confidence interval; GORD, gastro-esophageal reflux disease.

The ICU admittance and mechanical ventilator use incidence rates were presented by Kaplan–Meier analysis. COPD patients with GORD had a higher incidence rate of admission to the ICU than those without GORD. The incidence rate of admission to the ICU was 5.22 per 1,000 person-months in COPD patients with GORD, but only 3.01 per 1,000 person-months in the cohort without GORD. The log-rank test showed that patients with GORD had significantly higher incidence rates of ICU admission than those without GORD (*P* <0.0001) (Figure 2A). In addition, the Kaplan–Meier estimates of the 12-month incidence rate of mechanical ventilation were 4.34 per 1,000 person-months in the GORD cohort (95% confidence interval (CI), 3.38 to 5.58) and 2.41 per 1,000 person-months (95% CI, 1.90 to 3.05) for the comparison cohort (Figure 2B). The log-rank test revealed a statistically significant difference between the incidence rates over time (*P* = 0.0007).

### GORD is independently associated with ICU admittance and mechanical ventilator use in COPD patients

Table 2 presents a summary of the univariate Cox proportional hazards regression analyses. In addition to GORD, the factors age, CCI category, proxy COPD severity, and monthly insurance premium were associated with ICU admittance. The factors associated with mechanical ventilator use included GORD, age, occupational categories, and monthly insurance premium. The association between GORD and risk of ICU admission remained after adjusting for these factors in the multivariate analysis (adjusted hazard ratio, 1.75; 95% CI, 1.28 to 2.38, *P* <0.0001) (Table 3). Patients with GORD were independently associated with a 1.92-fold increased risk of mechanical ventilation use compared with comparison subjects (95% CI, 1.35 to 2.72; *P* <0.0001).

**Table 2:** **Crude hazard ratio of COPD patients’ serious events**

**Factor**	**ICU admittance**		**Ventilator used**	
	**Hazard ratio**	**95% CI**	**Hazard ratio**	**95% CI**
Without GORD	Reference		Reference	
With GORD	1.67	1.22 to 2.27^***^	1.85	1.31 to 2.62^***^
Male	1.12	0.8 to 1.56	1.18	0.81 to 1.72
Age 40 to 50	Reference		Reference	
Age 51 to 60	3.48	1.31 to 9.28^*^	1.48	0.58 to 3.76
Age 61 to 70	6.6	2.62 to 16.6^***^	3.64	1.62 to 8.2^***^
Age 71 to 80	9.92	4.00 to 24.6^***^	5.02	2.27 to 11.1^***^
Age ≥80	12.2	4.66 to 31.8^***^	8.99	3.9 to 20.7^***^
CCI = 0	Reference		Reference	
CCI = 1 to 3	1.77	0.24 to 12.9	1.59	0.22 to 11.6
CCI = 4 to 6	3.09	0.43 to 22.4	2.3	0.32 to 16.8
CCI ≥7	9.59	1.33 to 68.9^*^	7.13	0.99 to 51.5
Theophylline used	1.26	0.77 to 2.06	1.87	0.98 to 3.57
Proxy COPD severity	2.24	1.21 to 4.12^**^	1.48	0.65 to 3.37
Residential area				
Urban	Reference		Reference	
Suburban	1.32	0.95 to 1.82	1.55	1.08 to 2.23
Rural	0.89	0.50 to 1.60	1.14	0.61 to 2.12
Occupation categories				
Category 1	Reference		Reference	
Category 2	1.13	0.77 to 1.66	1.6	1.01 to 2.55^*^
Category 2	1.28	0.84 to 1.94	1.76	1.07 to 2.90^*^
Monthly insurance premium ($NT)^a^				
No fee	Reference		Reference	
1to 19,199	0.76	0.51 to 1.12	1.04	0.67 to 1.61
19,200 to 23,999	0.63	0.43 to 0.92^*^	0.77	0.5 to 1.2
≥ 24,000	0.29	0.15 to 0.56^***^	0.24	0.1 to 0.56^***^

CCI, Charlson Comorbidity Index; CI, confidence interval; COPD, chronic obstructive pulmonary disease; GORD, gastro-esophageal reflux disease; NT, new Taiwan dollar. *P <0.05, **P <0.01, ***P <0.0001. ^a^US$1 ≒ $NT30.

**Table 3:** **Adjusted hazard ratio of ICU admittance and mechanical ventilation**

**Factor**	**ICU admittance**		**Mechanical ventilation**	
	**HR**_**adj**_	**95% CI**	**HR**_**adj**_	**95% CI**
Without GORD	Reference		Reference	
With GORD	1.75	1.28 to 2.38^***^	1.92	1.35 to 2.72^***^

Adjusted for sex, age, Charlson Comorbidity Index category, proxy chronic obstructive pulmonary disease severity, occupational category, monthly insurance premium. CI, confidence interval; GORD, gastro-esophageal reflux disease; HR_adj_, adjusted hazard ratio. ***P <0.0001.

### Sensitivity analyses

**Figure 3:**
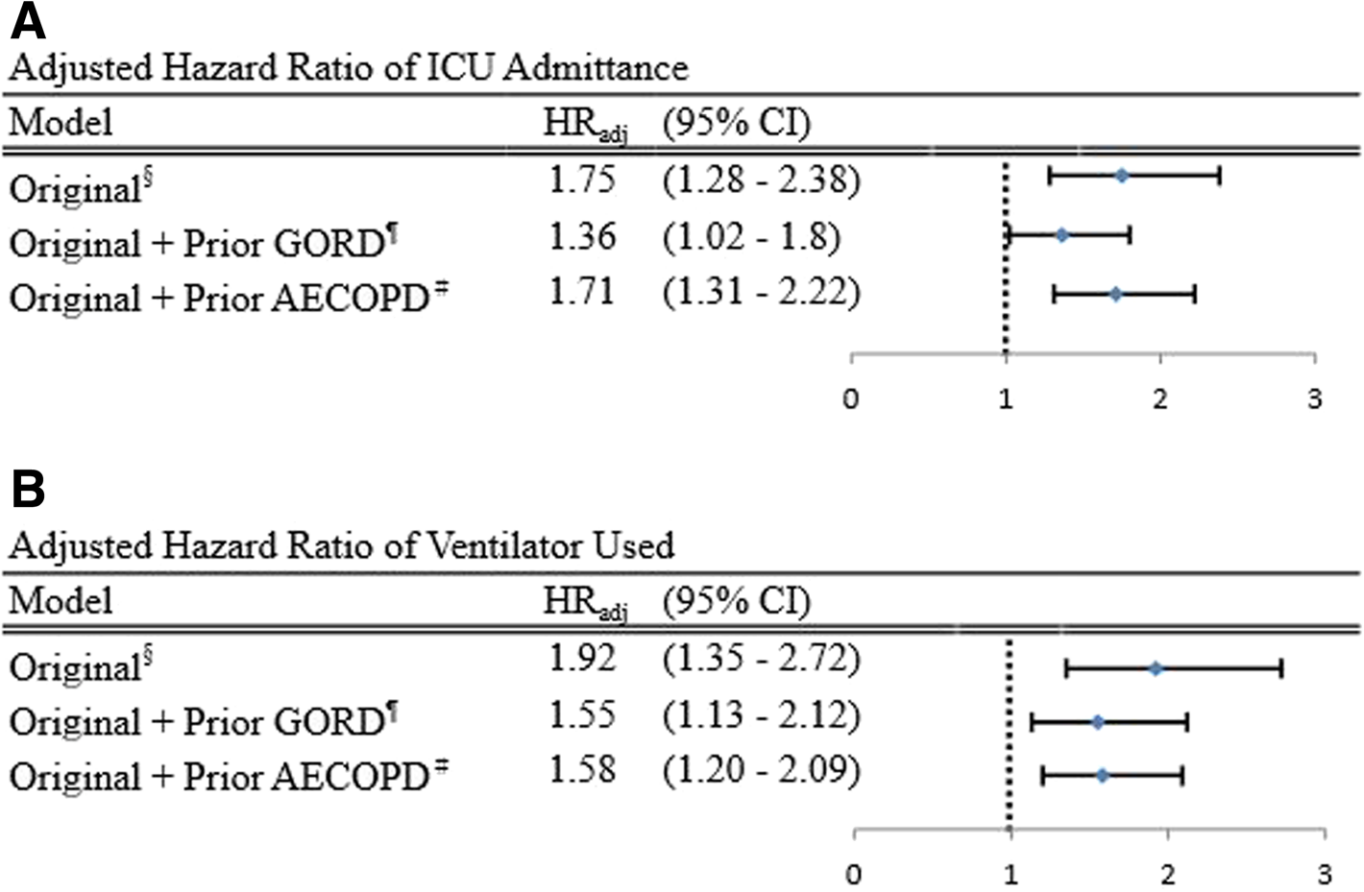
**Sensitivity analysis. (A)** Adjusted hazard ratio of ICU admittance. **(B)** Adjusted hazard ratio of ventilator used. ^§^Adjusted for sex, age, CCI category, proxy COPD severity, occupational category, and monthly insurance premium. ^¶^Adjusted for sex, age, CCI category, proxy COPD severity, occupational category, monthly insurance premium, and prior GORD. ^#^Adjusted for sex, age, CCI category, proxy COPD severity, occupational category, monthly insurance premium, and prior AECOPD. AECOPD, Acute exacerbation of chronic obstructive pulmonary disease; CCI, Charlson Comorbidity Index; CI, confidence interval; COPD, chronic obstructive pulmonary disease; GORD, gastro-esophageal reflux disease; HR_adj_, adjusted hazard ratio.

Figure 3 indicates that the increased risk of ICU admittance and ventilator use remained significant even after including people diagnosed with GORD prior to COPD and AECOPD within the first year preceding the index date. After including GORD prior to COPD as a covariate, GORD was found to be independently associated with a 1.36-fold (95% CI, 1.02 to 1.80) increased risk of ICU admittance and a 1.55-fold increased risk of ventilator use (95% CI, 1.13 to 2.13). In the analysis treating prior AECOPD as a covariate, GORD was also found to be independently associated with a 1.71-fold (95% CI, 1.31 to 2.22) increased risk of ICU admittance and a 1.58-fold increased risk of ventilator use (95% CI, 1.20 to 2.09).

## Discussion

This is the first investigation to detect a significantly higher incidence rate and independently increased risk of both admission to an ICU and mechanical ventilation use among COPD patients who subsequently developed GORD during the first year following their GORD diagnosis compared with COPD patients who did not develop GORD.

In the sensitivity analysis, we further demonstrated that a diagnosis of GORD after COPD is an independent risk factor for ICU admittance and mechanical ventilation even after including patients whose GORD was diagnosed prior to their COPD diagnosis and those patients that suffered an AECOPD within the first year preceding their diagnosis with GORD.

GORD is a frequently observed condition in patients with COPD [15,18,25,33]. GORD symptoms have been demonstrated to be an important factor associated with AECOPD (relative risk, 6.55) [18]. In our previous population-based study, we demonstrated newly developed GORD in COPD patients to be an independent risk factor for AECOPD [25]. However, not all AECOPD events are equal in terms of severity. Patients with COPD admitted to an ICU for an acute exacerbation have a substantial hospital mortality (24%) that has been associated with the development of nonrespiratory organ system dysfunction and exacerbation of the underlying respiratory disease [20]. While patients undergoing acute exacerbations resulting in respiratory failure or ICU admittance are at a high risk for poor outcomes, few studies in the literature have investigated the risk factors that may be associated with the severity of these exacerbations [34,35].

The only prior study utilizing large-scale data to explore the relationship between GORD and COPD including ICU admittance as an outcome of severity was performed in Korea by Kim and colleagues [15]. They sourced data from the National Health Insurance Database of Korea to conduct a cross-sectional study investigating the prevalence and risk factors of GORD in patients with COPD as well as the association between GORD and COPD exacerbation. They found that GORD was associated with an increased risk of AECOPD requiring hospitalization (odds ratio, 1.54; 95% CI, 1.50 to 1.58) and frequent emergency department visits (odds ratio, 1.55; 95% CI, 1.48 to 1.62). However, they did not detect an increased risk of ICU admission.

While Kim and colleagues did detect that there were significant differences in demographic characteristics, comorbidities, and pharmaceutical prescriptions between their COPD with and without GORD groups, they did not adjust for these parameters in their regression analysis [15]. The only factors they adjusted for were sex, age, type of health insurance, and COPD severity by pharmaceutical prescription. As some of these unadjusted factors are independent predictors for AECOPD, the results of that study were probably confounded. Moreover, as Kim and colleagues’ study was cross-sectional in design, they were unable to establish either temporality or a cause–effect relationship, and thus were further unable to report any measure of risk.

The current cohort study did adjust for demographic characteristics and medical comorbidities in our multivariate logistic regression analysis, only included newly diagnosed cases of GORD, and did exclude all the subjects who had ever received a diagnosis of asthma. This allowed us to better establish temporality, which is a necessary prerequisite to establishing a cause–effect relationship and critical to the elucidation of an underlying mechanism. This study also further excluded patients who had suffered an AECOPD in the year preceding study recruitment, which is a strong predictor for subsequent AECOPD and considered an indicator for the frequent exacerbator phenotype [14].

The estimates reported in this study may thus be considered independent and robust because of the above-mentioned inclusion and exclusion criteria as well as our comprehensive statistical adjustment. These results further underscore the association between GORD and worse outcomes among COPD patients, as well as demonstrating that even in a relatively stable group of COPD patients, a diagnosis of GORD is associated with an increased risk of severe outcomes, namely ICU admittance and mechanical ventilation use.

The pathological role of GORD in subjects with COPD is not conclusive. There are several mechanisms through which GORD may have proceeded pathologically to produce the increased risk of ICU admission and mechanical ventilator use observed in this study. As mentioned, the principal etiological factor precipitating the above outcomes is likely to be AECOPD, which in turn may have been brought on by the reflux of gastric contents into the esophagus of GORD patients. This would not have been experienced by the comparison cohort, and may help explain the differences observed between the two cohorts in this study. The first of these is the possibility of silent microaspiration, which would produce an inflammatory reaction [12,36] resulting in a heightened bronchial reactivity and increased airway resistance. Another possibility is that the anatomical changes leading to the flattening of the diaphragm seen in COPD patients may produce GORD symptoms. These changes may proceed through the loosening of the lower esophageal sphincter, which would allow gastric contents to reflux more easily [8] and augment airway hyperresponsiveness through the vagal reflex [36,37].

The results of this study may suggest that GORD-like symptoms are an extrapulmonary manifestation of COPD and are warning signs for disease progression. Prior work has demonstrated that ventilated patients experience difficulty weaning due to a reduction in splanchnic blood flow [38]. Thus, it is also possible that a reduction in splanchnic blood flow might also result in symptoms similar to GORD in COPD patients. These symptoms might arise from the gradual exacerbation of dyspnea and increase in breathing workload among COPD patients, which in turn engender an augmented intra-abdominal pressure, thus working to further reduce oxygen delivery to splanchnic organs [39]. As GORD-like symptoms occurring in COPD patients may be a product of the pathophysiological changes experienced by COPD patients, such symptoms may thus be useful as a warning sign for disease progression and warrant further investigation.

However, COPD patients may be admitted to the ICU for many reasons in addition to respiratory failure, and while we used multiple strategies to ensure for the adequateness of the study population and to minimize the effect of confounding factors and competing etiologies, there still probably remain other comorbid conditions and mechanisms at play. Nevertheless, while our study is the first to identify the prospective impact of GORD on ICU admission and mechanical ventilator use among COPD patients, our results need to be interpreted through a number of limitations.

First, we were unable to obtain information regarding the real severity of airflow obstruction, clinical symptoms, or Body-mass index, airflow Obstruction, Dyspnea, and Exercise (BODE) index. We recommend that prospective studies clarify the effect these factors have on the associations detected in this study.

Second, the diagnoses sourced in this study were based on ICD-9-CM codes and might not be as reliable as those made according to the well-defined criteria of prospective studies. However, to help assure for diagnostic validity, we only included COPD subjects that had received two diagnoses and were prescribed medication.

Third, comorbidities were quantified according to the CCI. This index assigns a score to each disease that is proportionate to the disease-related risk of death. The arithmetic sum of scores of individual diseases coexisting in the same patient provides the index of comorbidity. Neither indices of COPD severity nor comorbid conditions other than the components of the CCI were available. However, this index has been developed to predict mortality among patients with chronic diseases, and has been widely used and validated for most major diseases including COPD [40-43]. Moreover, we established a severity indicator in accordance with the latest GOLD guidelines to adjust for the severity of COPD in our multivariate analysis.

## Conclusion

This study is the first investigation to detect a significantly higher incidence rate and independently increased risk of both admission to an ICU and mechanical ventilation use among COPD patients who subsequently developed GORD during the first year following their GORD diagnosis compared with COPD patients who did not develop GORD. The authors hope that these results will encourage physicians treating COPD patients with GORD to exercise caution and be cognizant of their increased risk for severe outcomes.

## Key messages

- GORD symptoms are an important risk factor associated with AECOPD, which is the principal etiological factor precipitating the majority of unscheduled visits and hospitalizations for patients with COPD.
- We have demonstrated that COPD patients with GORD have a higher incidence rate of admission to the ICU and mechanical ventilation use than those without GORD during the first year following their GORD diagnosis than COPD patients without GORD.
- After weighting comorbidities by propensity score, COPD patients diagnosed with GORD had a significantly increased risk of admission to the ICU (hazard ratio, 1.75) and mechanical ventilation use (hazard ratio, 1.92) within the first 12 months following a diagnosis of GORD.
- We have established temporality in the relationship between COPD and GORD. Caution should be exercised when assessing GORD symptoms among COPD patients in clinical practice. Physicians should be cognizant of their increased risk for severe outcomes.

## Additional files

Additional file 1:
**is a table presenting the ICD-9-CM code definitions for diagnoses.**
Additional file 2:
**is a table presenting the occupation categories.**

## Abbreviations

AECOPD: acute exacerbation of chronic obstructive pulmonary disease
CCI: Charlson Comorbidity Index
CI: confidence interval
COPD: chronic obstructive pulmonary disease
GOLD: Global Initiative for Chronic Obstructive Lung Disease
GORD: gastro-esophageal reflux disease
ICD-9-CM: International Classification of Diseases, 9th revision, Clinical Modification
NHIRD: National Health Insurance Research Database
