# Supplementary material for: Gastro-oesophageal reflux disease increases the risk of intensive care unit admittance and mechanical ventilation use among patients with chronic obstructive pulmonary disease: a nationwide population-based cohort study
Source: Crit Care. 2015 Mar 24;19(1):110. doi: 10.1186/s13054-015-0849-1 (PMC4422143; doi:10.1186/s13054-015-0849-1)
Supplement: Additional file 1: — is a table presenting the ICD-9-CM code definitions for diagnoses. [file 13054_2015_849_MOESM1_ESM.pdf]

Additional file 1 ICD-9-CM code Definitions for Diagnoses

Diagnosis	ICD-9-CM code
COPD	491, 492, 496
GERD	530.11, 530.81, 530.10
Asthma	493
Lung Cancer	162~164
Disease of esophagus	750.4, 530.3
Dyskinesia of esophagus	530.5
Malignant neoplasm of esophagus	150
Zollinerger-Ellison syndrome	251.5
Alcohol dependence syndrome	303
Obesity	278
Myocardial Infarction	410, 412
Congestive Heart Failure	428
Periphral Vascular Disease	441,443.9,785.4, V 434
Cerebrovascular Disease	430,431,432,433,434,435,436,437,438
Dementia	290
Pulmonary Disease	490, 494,495, 500,501,502, 503, 504, 505
Connective Tissue Disorder	710.0, 710.1, 710.4, 714.0, 714.1, 714.2 , 714.81, 517.1, 725
Peptic Ulcer	531, 532, 533, 534
Liver Disease	250.0, 250.1, 250.2, 250.3, 250.7
Diabetes complications	250.4, 250.5, 250.6
Paraplegia	342, 344.1
Renal Disease	582, 583.0, 583.1, 583.2, 583.3, 583.4, 583.5, 583.6, 583.7, 585, 586, 588
Cancer	14, 15, 16, 170, 171, 172, 174, 175, 176, 179, 18, 190, 191, 192, 193, 194, 195.0, 195.1, 195.2, 195.3, 195.4, 195.5, 195.8, 200, 201, 202, 203, 204, 205, 206, 207, 208
Metastatic cancer	196, 197, 198, 199.0, 199.1
Severe liver disease	572.2, 572.3, 572.4, 572.8
AIDS/HIV	042, 043, 044
