# Supplementary material for: Gastro-oesophageal reflux disease increases the risk of intensive care unit admittance and mechanical ventilation use among patients with chronic obstructive pulmonary disease: a nationwide population-based cohort study
Source: Crit Care. 2015 Mar 24;19(1):110. doi: 10.1186/s13054-015-0849-1 (PMC4422143; doi:10.1186/s13054-015-0849-1)
Supplement: Additional file 2: — is a table presenting the occupation categories. [file 13054_2015_849_MOESM2_ESM.pdf]

**Additional file 2** Occupation categories:

| Category | Occupation |
|---|---|
| Category 1 | Civil services, Institution workers, Enterprise, Business, and Industrial administration personnel |
| Category 2 | Farmers, Fishermen, Vendors, and Industrial laborers |
| Category 3 | All other occupations |
